# Investigation of the role of tyrosine kinase receptor EPHA3 in colorectal cancer

**DOI:** 10.1038/srep41576

**Published:** 2017-02-07

**Authors:** Elena Andretta, Fernando Cartón-García, Águeda Martínez-Barriocanal, Priscila Guimarães de Marcondes, Lizbeth M. Jimenez-Flores, Irati Macaya, Sarah Bazzocco, Josipa Bilic, Paulo Rodrigues, Rocio Nieto, Stefania Landolfi, Santiago Ramon y Cajal, Simo Schwartz, Arthur Brown, Higinio Dopeso, Diego Arango

**Affiliations:** 1 Group of Biomedical Research in Digestive Tract Tumors, CIBBIM-Nanomedicine, Vall d’Hebron University Hospital, Research Institute (VHIR), Universitat Autònoma de Barcelona, Passeig Vall d’Hebron, 119-129, 08035 Barcelona, Spain; 2 Department of Pathology, Vall d’Hebron Hospital, Barcelona, Spain; 3 Group of Drug Delivery and Targeting, CIBBIM-Nanomedicine, Vall d’Hebron University Hospital, Research Institute (VHIR), Universitat Autònoma de Barcelona, Barcelona, Spain; 4 CIBER de Bioingeniería, Biomateriales y Nanomedicina (CIBER-BBN), Spain; 5 Robarts Research Institute, London, Ontario, Canada

## Abstract

EPH signaling deregulation has been shown to be important for colorectal carcinogenesis and genome-wide sequencing efforts have identified *EPHA3* as one of the most frequently mutated genes in these tumors. However, the role of EPHA3 in colorectal cancer has not been thoroughly investigated. We show here that ectopic expression of wild type EPHA3 in colon cancer cells did not affect their growth, motility/invasion or metastatic potential *in vivo*. Moreover, overexpression of mutant EPHA3 or deletion of the endogenous mutant EPHA3 in colon cancer cells did not affect their growth or motility. EPHA3 inactivation in mice did not initiate the tumorigenic process in their intestine, and had no effects on tumor size/multiplicity after tumor initiation either genetically or pharmacologically. In addition, immunohistochemical analysis of EPHA3 tumor levels did not reveal associations with survival or clinicopathological features of colorectal cancer patients. In conclusion, we show that EPHA3 does not play a major role in colorectal tumorigenesis. These results significantly contribute to our understanding of the role of EPH signaling during colorectal carcinogenesis, and highlighting the need for detailed functional studies to confirm the relevance of putative cancer driver genes identified in sequencing efforts of the cancer genome.

Since the first EPH receptor was cloned over 25 years ago^1^ this family has expanded and become the largest family of receptor tyrosine kinases, with at least 9 type A and 5 type B EPH receptors. EPH receptors are classified into the A and B subclass depending on sequence similarity and the type of Ephrin ligands that they bind^2^. Ephrin A and B ligands are anchored to the plasma membrane either through a glycosyl-phosphatidylinositol (GPI) moiety, or a transmembrane domain, respectively. These receptors and their Ephrin ligands are important for recognizing signals from the extracellular environment and are involved in cell-cell interaction, participating in cell adhesion, migration and proliferation, both during embryogenesis and in adult tissues^2^,^3^,^4^. In the normal intestinal epithelium, EPHB2 and EPHB3 have a key role in maintaining spatially separate proliferative and differentiating compartments^5^,^6^. Moreover, abrogation of EPHB2 and EPHB3 signaling reduces the number of proliferating cells in intestinal crypts by >50%^7^.

Deregulation of EPH signaling has been shown to be important for tumor progression in different organs, including colorectal tumors^2^. The loss of EPHB2, EPHB3 and EPHB4 significantly contributes to the early stages of the tumorigenic process^8^,^9^,^10^,^11^. Despite the documented relevance of the inactivation of these EPH receptors on the oncogenic process of colorectal tumors, genome-wide sequencing efforts did not identify recurrent mutations in these receptors or in their ligands^12^,^13^,^14^,^15^. Surprisingly, however, *EPHA3* was identified in early genome-wide sequencing studies in colorectal tumors^14^ as the sixth most frequently mutated gene with mutations in >11% of the tumors and a cancer mutation prevalence (CaMP) score >4. Only *APC, TP53, KRAS, FBXW7* and *SMAD4* showed CaMP scores higher than *EPHA3* and the mutation frequency in this EPH receptor was higher than the incidence observed in other known tumor suppressor genes in this organ, such as *SMAD2* and *TGFBR2*^14^. Moreover, additional studies have confirmed the presence of recurrent *EPHA3* mutations in colorectal tumors^12^,^13^ that are distributed throughout the coding sequence of *EPHA3* (Supplementary Figure 1) and that have been shown to disrupt EPHA3 signaling^16^. Based on these genetic data, and the known importance of EPH signaling in colorectal cancer, EPHA3 inactivation would be expected to significantly contribute to the progression of colorectal tumors. However, the role of EPHA3 in the oncogenic process of this organ has not been investigated.

Here we used inducible isogenic cell line systems, animal models and large human tumor collections to investigate the role of EPHA3 during colorectal cancer progression. Despite the evidence from the genetic screens, we found that overexpression of wild type or mutant EPHA3 did not affect the growth of colon cancer cells. In addition, targeted inactivation of EphA3 in mice did not initiate the tumorigenic process in the intestinal epithelium, and when tumors were initiated genetically or pharmacologically, EphA3 inactivation had no effects on animal survival, tumor size or multiplicity. Moreover, reintroduction of EPHA3 in colon cancer cells did not change their motility/invasion *in vitro* or their metastatic potential in a mouse model of experimental metastasis. Finally, immunohistochemical analysis revealed that the levels of EPHA3 expression in colorectal tumors were not associated with any clinicopathological features or with patient survival. Collectively, our results indicate that EPHA3 inactivation is not an important step in the tumorigenic process of colorectal tumors, and highlights the need to functionally validate the findings of large exome/genome sequencing studies.

## Results

### Generation of colon cancer cell lines with inducible EPHA3 activity

**Figure 1:**
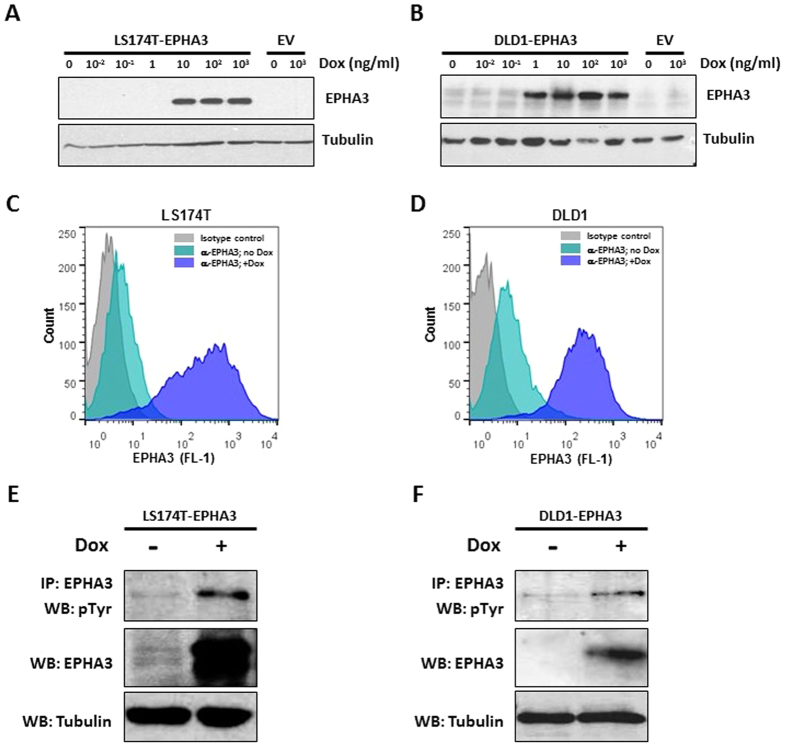
Inducible EPHA3 overexpression in colon cancer cell lines. (**A**,**B**) Western blot showing the levels of EPHA3 protein in LS174T (**A**) and DLD1 (**B**) cells stably transfected with pLenti/TO-EPHA3 or the control empty vector (EV) after treatment with the indicated concentrations of doxycycline for 48 h. Tubulin levels are shown as a loading control. (**C**,**D**) Cell surface levels of EPHA3 following induction with doxycycline (1 μg/ml; 48 h) were assessed by flow cytometry analysis in LS174T-EPHA3 (**C**) and DLD1-EPHA3 (**D**). (**E**,**F**) The levels of EPHA3 phosphorylation after doxycycline treatment (1 μg/ml) for 48 h were determined by immunoprecipitation with anti-EPHA3 and Western blotting with anti-phospho-Tyrosine. Total input levels of EPHA3 and tubulin are also shown for LS174T-EPHA3 (**E**) and DLD1-EPHA3 (**F**).

Several EPH receptors and their Ephrin ligands have been found to be aberrantly expressed in multiple cancer types and to significantly contribute to the oncogenic process of colorectal tumors^8^,^11^,^17^. To investigate the functional role of EPHA3 in colorectal cancer, we engineered two cell line systems with doxycycline-dependent inducible EPHA3 expression. LS174T and DLD1 colon cancer cell lines were used because they express low endogenous levels of wild type or compound heterozygous mutant (T719I and M847K) EPHA3 protein^18^, respectively (Supplementary Figure 2A), and high levels of the preferred EPHA3 ligand EphrinA5 (Supplementary Figure 2B). These cell lines were stably transfected with a vector expressing wild type human EPHA3 under the control of the doxycycline-inducible CMV/TO promoter (pLenti-CMV/TO-EPHA3) or the corresponding control empty vector. Doxycycline-dependent expression of EPHA3 was confirmed on individual clones by Western blot (Fig. 1A,B) and membrane localization was assessed by flow cytometry analysis (Fig. 1C,D). Moreover, phosphorylation of the EPHA3 receptor upon doxycycline treatment was confirmed by an immunoprecipitation assay, thus confirming activation of the EPHA3 signaling pathway in these cells (Fig. 1E,F).

### EPHA3 does not regulate the growth of colon cancer cells

**Figure 2:**
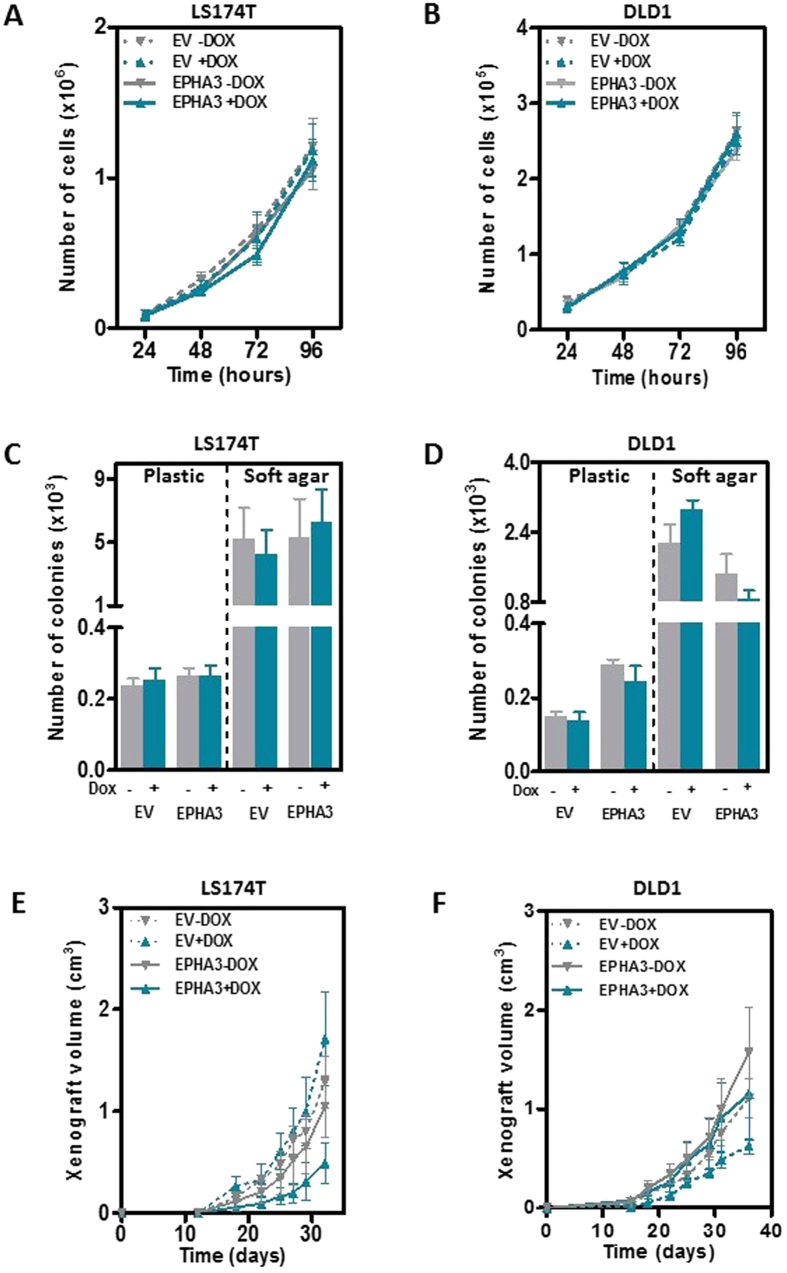
Effects of EPHA3 overexpression on the growth of colon cancer cells. (**A**,**B**) The average number of LS174T-EPHA3 (**A**) and DLD1-EPHA3 (**B**) cells and the corresponding empty vector control (EV) at the indicated times after seeding is shown (mean ± SEM of three independent experiments). (**C**,**D**) Number of colonies of LS174T-EPHA3 (**C**) and DLD1-EPHA3 (**D**) cells and the corresponding empty vector control (EV) grown on a solid plastic substrate (left), or on soft agar (right) with or without doxycycline treatment (1 μg/ml). The mean ( ±SEM) of three independent experiments run in triplicate is shown. (**E**,**F**) Growth of LS174T-EPHA3 (**E**) and DLD1-EPHA3 (**F**) cells when injected subcutaneously in immunodeficient NOD/SCID mice. Doxycycline was administered to the indicated groups of animals in the drinking water. The average tumor size ( ±SEM) is shown. N = 6 animals per group.

EPH signaling has been shown to be important to sustain the active proliferation rates found in the intestinal epithelial cells and in intestinal tumors^8^,^9^,^10^,^11^. Here we used the cell line systems generated to study whether EPHA3 signaling regulates the proliferative activity of colon cancer cells. First, we investigated whether the reintroduction of EPHA3 into LS174T and DLD1 colon cancer cells modulated their growth *in vitro* by directly counting the number of cells at different times post-seeding, and found no differences after EPHA3 overexpression by doxycycline treatment (Fig. 2A,B). Moreover, ectopic expression of wild type EPHA3 did not affect the capacity of colon cancer cells to form colonies when growing on a solid or a semisolid soft-agar substrate (Fig. 2C,D). The effects of EPHA3 on the growth of colon cancer cells were further investigated using a xenograft model. LS174T-EPHA3 or control LS174T-EV were injected subcutaneously in the right and left flank of NOD/SCID immunodeficient mice, respectively. Animals were then randomized to receive doxycycline in the drinking water or a control group, and xenograft growth was monitored over time. The same experimental layout was carried out with the DLD1-EPHA3 derivative lines. No differences were observed in the average size of the xenografts formed by these cell lines in animals treated with doxycycline compared to untreated control mice (Fig. 2E,F). EPHA3 overexpression was confirmed by immunohistochemistry at the end of the experiment in the tumors from doxycycline-treated mice (Supplementary Figure 3). Collectively, these results show that the reintroduction of EPHA3 into deficient colon cancer cells does not affect their growth.

### Characterization of the role of Epha3 in intestinal tumorigenesis using a knockout mouse model

Because other EPH receptors have been shown to be involved in the oncogenic process of intestinal tumors using mouse models^8^,^11^, we next decided to investigate the role of this Eph receptor *in vivo* using a knockout mouse model where the first exon of *EphA3* has been deleted by homologous recombination^19^. We confirmed loss of expression in the intestine and liver of *Epha3* KO mice by quantitative real-time RT-PCR (Supplementary Figure 4). As previously reported, we observed perinatal mortality of approximately two thirds of the *EphA3*^*−/−*^ mice due to cardiac defects, although the remaining 34% of the knockout mice were viable with no obvious cardiac phenotype or any other abnormalities^19^,^20^.

**Figure 3:**
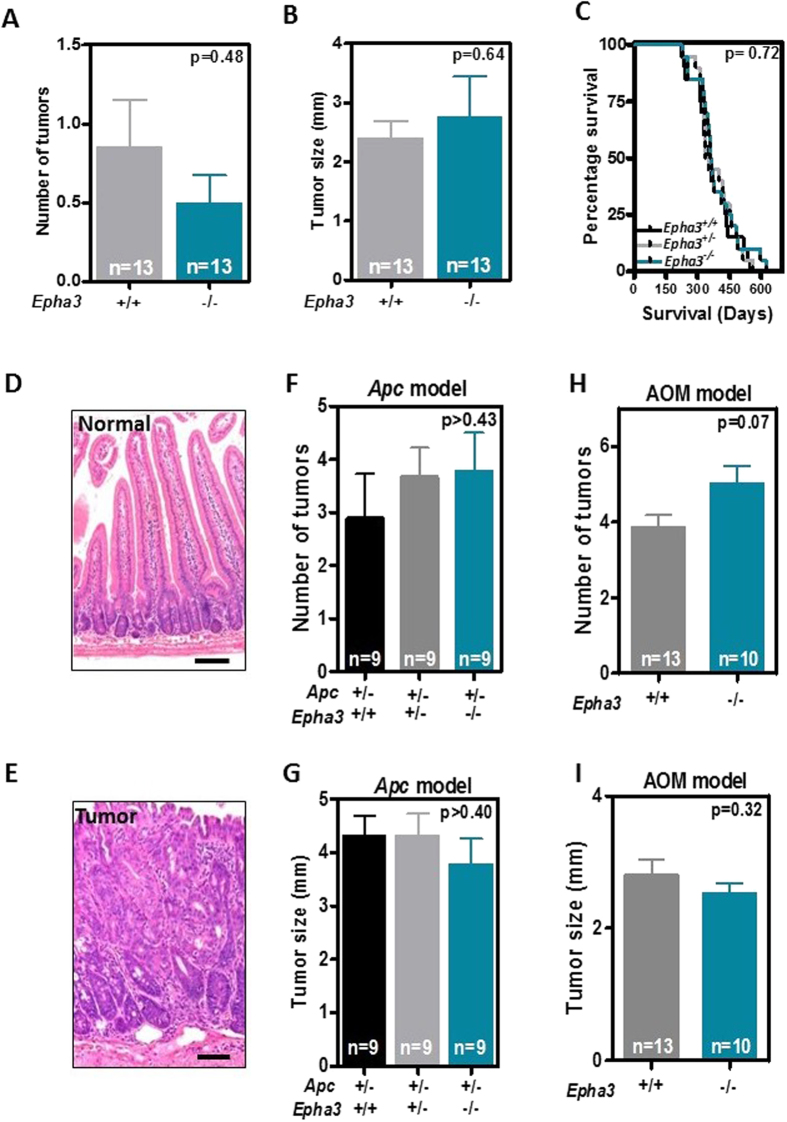
Effects of targeted inactivation of *Epha3* on mouse intestinal tumorigenesis. (**A**,**B**) Histograms showing the number (**A**) and size (**B**) of intestinal tumors in *EphA3* wild type and knockout mice at the age of 20 months. (**C**) Survival of *Apc*^*min/+*^ mice that are either wild type, heterozygous or homozygous for *EphA3* inactivation (n = 20 animals per group). (**D**) Representative histological image of the normal small intestine, and (**E**) a representative small intestinal tumor. Samples were stained with hematoxylin and eosin. Scale bar 100 μm. (**F**,**G**) Number (**F**) and size (**G**) of small intestinal tumors observed in 41-week-old *Apc*^*min/+*^ mice that are either wild type, heterozygous or homozygous for *EphA3* inactivation. (**I**,**J**) Number (**I**) and size (**J**) of large intestinal tumors observed in 25-week-old wild type or *EphA3* knockout mice after azoxymethane (AOM) treatment. N = number of animals per group. All histograms show average values ± SEM. All p-values shown were calculated using Student’s T-test.

Importantly, we found that the loss of Epha3 does not affect adult animal survival (Supplementary Figure 5) or the incidence of intestinal tumors at 20 months of age (Fig. 3A,B), indicating that the loss of Epha3 does not promote tumor initiation. Intestinal tumorigenesis was therefore initiated genetically by crossing the *EphA3* model with *Apc*^*min/+*^ mice carrying heterozygous mutations in the *Apc* tumor suppressor gene^21^. We observed that the lifespan of *Apc*^*min/+*^ mice was not affected by the loss of one or two copies of *EphA3* (Fig. 3C). In good agreement with this finding, the number, size or histological type of intestinal tumors at 41 weeks of age was not different in *Apc*^*min/+*^ mice that are either wild type, heterozygous or homozygous for the *EphA3* knockout allele (Fig. 3D–G). The majority of the intestinal tumors observed were adenomas (54 of 83; 65.1%). Some adenocarcinomas that invaded through the mucosa (20.5%), submucosa (4.8%) or the muscularis propria (6.0%) were also observed. In addition, intestinal tumorigenesis was induced pharmacologically in an independent cohort of mice with the intestinal-specific carcinogen azoxymethane (AOM). However, consistent with the findings of the *Apc*^*min/+*^ model, no differences were observed in the number, size or histology of large intestinal tumors in *EphA3* wild type and knockout mice (Fig. 3H,I). Most tumors found were adenomas (25 of 36; 69.4%). Some adenocarcinomas infiltrating the mucosa (25.0%) or the submucosa (5.6%) were also observed in the large intestine, while no tumors were found in the small intestine of AOM-treated mice. Overall, these experiments indicate that, unlike the loss of EphB receptors, EphA3 inactivation does not significantly contribute to intestinal tumor initiation or progression during the early stages of the tumorigenic process in murine models.

### Reintroduction of EPHA3 into deficient colon cancer cells does not affect their metastatic potential

**Figure 4:**
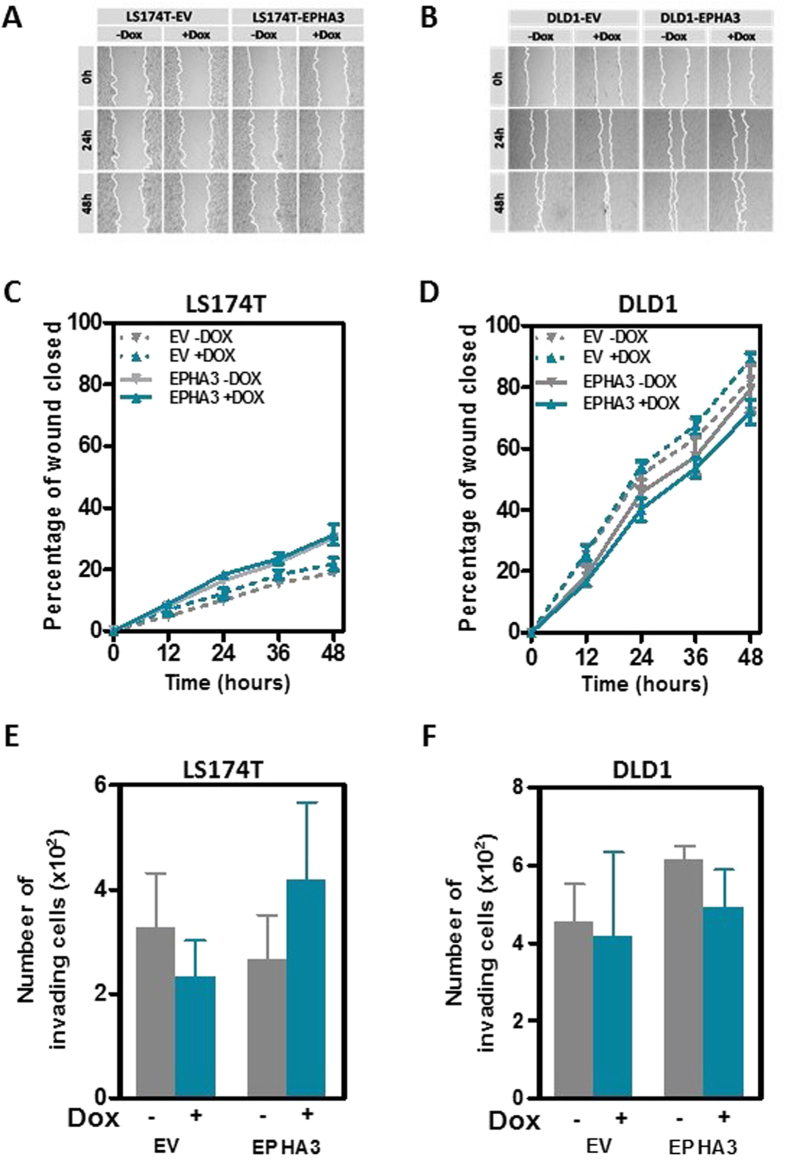
Effects of EPHA3 on colon cancer cell motility and invasion. (**A–D**) Changes in the motility of LS174T (**A** and **C**) and DLD1 (**B** and **D**) cells after EPHA3 overexpression were assessed using a wound healing assay. Cells transfected with the control empty vector (EV) were used along with cells expressing EPHA3 after doxycycline (Dox) treatment (1 μg/ml). Panels (**A**,**B**) show representative images and panels (**C**,**D**) show the average ( ±SEM) percentage of the initial wound closed after the indicated times in three independent experiments carried out in triplicate. (**E**,**F**) Matrigel Invasion capacity (Boyden chamber invasion assay) of LS174T-EPHA3 **(E)** and DLD1-EPHA3 **(F)** with and without doxycycline (Dox)-dependent induction of EPHA3 overexpression. The corresponding empty vector (EV) derivative lines were used to control for possible effects of doxycycline on the invasion of these cells. The average ( ±SEM) of three independent experiments carried out in triplicate is shown.

**Figure 5:**
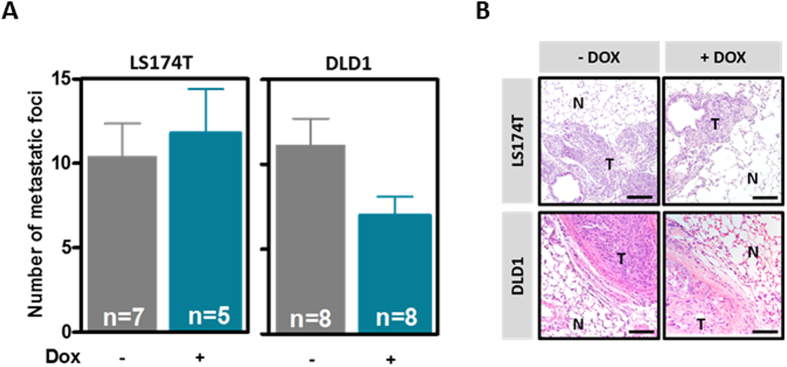
Effects of EPHA3 overexpression on the metastatic potential of colon cancer cells. (**A**) Average number ( ±SEM) of macroscopically visible metastases formed by LS174T-EPHA3 or DLD1-EPHA3 cells 6 or 10 weeks, respectively, after tail vein injection in NOD/SCID immunodeficient animals receiving doxycycline (Dox) in the drinking water or control animals. N = number of animals. (**B**) Representative images of hematoxylin and eosin stained histological lung sections of the mice in. N: normal; T: tumor. Scale bar: 100 μm.

Although our results with the *EphA3* knockout mouse model indicate that the loss of EphA3 is not an important event in the early stages of tumor development, it remained possible that EPHA3 may be involved in the metastatic process of colon cancer cells. Because EPHA3 has been shown before to regulate cell adhesion and migration in other tumor types^22^,^23^, we first investigated the effects of reintroducing wild type EPHA3 on the motility/migration capacity of colon cancer cells. A wound-healing assay demonstrated that EPHA3 overexpression did not change the motility of LS174T and DLD1 cells (Fig. 4A–D). Next, we investigated whether restoration of EPHA3 signaling affected the invasive potential of colon cancer cells. A Boyden chamber assay demonstrated that EPHA3 activation in LS174T and DLD1 cells did not affect the capacity of these cells to invade through matrigel (Fig. 4E,F). We then used an experimental model of lung metastasis where LS174T-EPHA3 or DLD1-EPHA3 cells were injected in immunodeficient NOD/SCID mice that were then randomized to a control group or a group receiving doxycycline in the drinking water to induce EPHA3 expression. No differences were observed in the number of lung metastases formed by LS174T or DLD1 cells after EPHA3 overexpression (Fig. 5A). The presence of metastatic lesions in the lungs of these animals was confirmed on histological sections (Fig. 5B). When considered together these results demonstrate that the reintroduction of EPHA3 in colon cancer cells does not interfere with their metastatic potential.

### *EPHA3* mutations do not affect proliferation or motility of colon cancer cells

**Figure 6:**
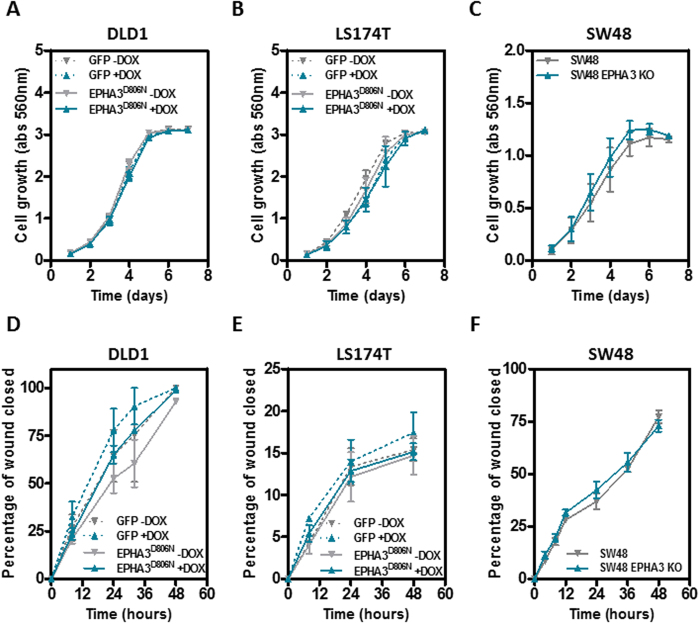
Effects of the modulation of mutant EPHA3 expression on proliferation and motility of colon cancer cells. Doxycycline-dependent overexpression of mutant EPHA3^D806N^ in DLD1 cells (**A**,**D**) and LS174T cells (**B**,**E**) or targeted deletion of the endogenous mutant EPHA3 in SW48 cells (**D**,**F**), did not affect cell growth (sulforhodamine B staining; **A–C**) or motility (wound healing assay; **D–F**). The average (±SEM) of three independent experiments carried out in triplicate is shown.

Collectively, our results indicate that wild type EPHA3 does not regulate the growth or metastasis of colorectal tumors. We next investigated whether the EPHA3 mutations frequently observed in colorectal tumors are oncogenic. First, we used a doxycycline-dependent inducible system to overexpress EPHA3^D806N^ in DLD1 and LS174T colon cancer cells (Supplementary Figure 6A–D). This mutation has been observed in skin and colon tumors^14^,^24^ and codes for a stable protein that lacks kinase activity^16^,^25^,^26^. In addition, we used a CRISPR/Cas9 approach to knockout the endogenous compound heterozygous mutant *EPHA3* in SW48 cells (K365Nfs*6 and G784E; Supplementary Figure 2A)^18^,^27^. Cells were transiently transfected with a vector expressing Cas9/GFP and a guiding RNA targeting the first exon of *EPHA3*. Three individual clones derived from GFP-positive cells were amplified and the presence of truncating mutations confirmed by direct sequencing and the loss of EPHA3 protein confirmed by Western blot (Supplementary Figure 6E,F). Three additional clones showing no EPHA3 mutations were used as control (Supplementary Figure 6E,F). Overexpression of the *EPHA3*^*D806N*^ mutant protein in DLD1 or LS174T cells, or deletion of the endogenous mutant *EPHA3* in SW48 cells had no effect on cell proliferation (Fig. 6A–C) or motility (Fig. 6D–F), indicating that these EPHA3 mutations observed in colon tumors do not confer a growth/motility advantage.

### Survival of colorectal cancer patients as a function of EPHA3 tumor levels

**Figure 7:**
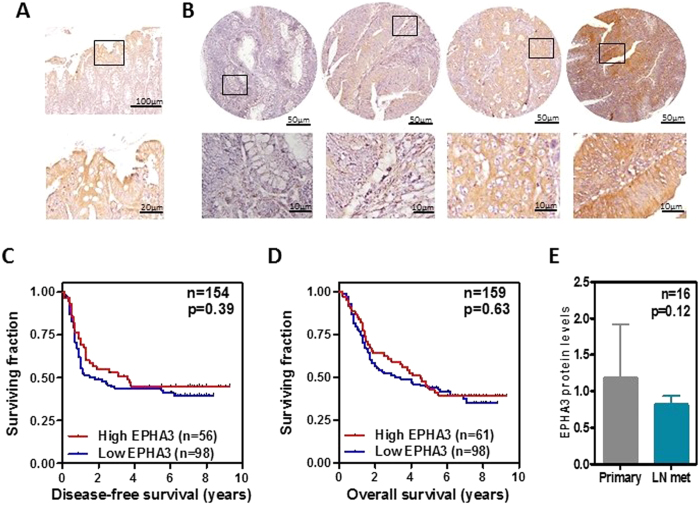
Survival of colorectal cancer patients with high and low EPHA3 levels in their tumors. The levels of EPHA3 protein were assessed by immunohistochemistry with a specific antibody (see Supplementary Figure 3) in normal colonic mucosa (**A**) and colorectal tumors (**B**). Disease-free (**C**) and overall survival (**D**) of 159 Dukes C colorectal cancer patients as a function of tumor EPHA3 levels was studied. P values are from the Logrank test. (**E**) Average protein expression levels in 16 paired primary Dukes C tumors and lymph node metastases from the same patients (p value shown is from a paired Student’s T-test).

An association has been observed between EPHA3 levels and the survival of patients with different tumor types, including colorectal cancer^25^,^28^,^29^,^30^,^31^. Here, we used a tissue microarray containing triplicate tumor samples from 159 patients with Dukes C colorectal cancer to investigate possible associations between EPHA3 levels and survival of colorectal cancer patients (Supplementary Table 1). The levels of EPHA3 protein expression in these tumors were determined by immunohistochemistry and the specificity of the antibody used was confirmed on formalin-fixed, paraffin-embedded samples from the xenografts generated with the cell lines engineered to overexpress EPHA3 (Supplementary Figure 3). Tumor samples showed variable levels of EPHA3 expression that ranged from undetectable to high expression (Fig. 7A,B). However, no associations were observed between EPHA3 expression and disease-free or overall survival (Logrank test p > 0.39; Fig. 7C,D) and other clinicopathological features including patient age, gender, tumor location and histological grade (Supplementary Table 1). In addition, for 16 of these primary Dukes C tumors, the paired lymph node metastasis from the same patient was also available. Consistent with our *in vitro* data and the model of experimental metastasis, no significant differences in the levels of EPHA3 were observed between matched primary and metastatic lesions (Fig. 7E), further indicating that EPHA3 does not regulate the metastatic dissemination of colon cancer cells.

## Discussion

EPH signaling has been shown to regulate the proliferative activity in the normal intestinal epithelium and maintain proliferating cells confined to the crypts of Lieberkühn^7^,^32^. In addition, disruption of EPH signaling has been reported to significantly contribute to the progression of colorectal cancer. We have previously reported frequent (>40%) *EPHB2* mutations in colorectal tumors with microsatellite instability^10^, and targeted inactivation of both alleles of *EphB2* or *EphB3*, or a single allele of *EphB4*, significantly accelerate the tumorigenic process initiated by *Apc* mutations in mouse models^8^,^33^. Moreover, the expression of several EPH receptors, including EPHB2, EPHB3, EPHB4 or EPHA2 are associated with the prognosis of patients with colorectal cancer^9^,^32^,^33^,^34^,^35^,^36^. Pioneer genome-wide exome sequencing studies reported that 11.4% (4/35) of colorectal tumors had mutations in *EPHA3*^14^ and these mutations have been reported to disrupt the function of EPHA3^16^, suggesting that they contribute to tumor progression. However, significantly lower *EPHA3* mutation frequency was observed in an extended cohort of colorectal tumors (4.05%; 42/1035)^37^,^38^,^39^. Interestingly, in lung cancer, where EPHA3 has been shown to have tumor suppressor activity^25^, the reported mutation frequency is over 9% (105/1144)^40^.

Here, we show that reintroduction of this EPH receptor into colon cancer cells with low levels of either wild type (LS174T) or mutant (**DLD1**) *EPHA3* and that express the preferred EPHA3 ligand (Ephrin A5), has no effects on the growth of cancer cells on a solid substrate, soft agar or when grown as subcutaneous xenografts in immunodeficient NOD/SCID mice. Consistently, we used a mouse model with targeted *EphA3* inactivation and demonstrated that the loss of EphA3 did not initiate the tumorigenic process in the intestine, and had no effects on the tumorigenic process initiated by heterozygous *Apc* mutations (*Apc*^*min/+*^ mice) or pharmacologically with the intestine-specific carcinogen azoxymethane (AOM). Therefore, despite the reported role of type B EPH receptors in the progression of colorectal cancer and the high frequency of EPHA3 mutations observed in early genome-wide mutatome analyses, our results indicate that modulation of EPHA3 signaling does not regulate the growth of intestinal cancer cells.

The intestinal tumors observed in the *Apc*^*min/+*^ and AOM animal models used do not metastasize and are therefore not ideally suited to investigate the role of EPHA3 on the metastatic dissemination of colorectal cancer cells. However, EPHA3 has been shown to regulate the attachment of melanoma cancer cells^23^ and is associated with metastatic dissemination in hepatocellular carcinoma^29^. Moreover, EPHA3 has been suggested to regulate the metastatic spread of colorectal tumors to lymph nodes and distant organs^31^,^41^. We therefore decided to further investigate the role of EPHA3 in the invasive and metastatic potential of colorectal tumors and found that reintroduction of EPHA3 in colon cancer cells had no effect on their motility/invasion *in vitro*. Consistently, reintroduction of EPHA3 in colon cancer cells did not affect their metastatic potential in an experimental model of lung metastasis and no differences were observed in the expression of EPHA3 in primary Dukes C tumors and matched lymph node metastases, indicating that EPHA3 does not play a major role in the metastatic process of these tumors. These results suggest that the role of EPHA3 on the metastatic potential of cancer cells is context dependent, contributing to disease dissemination in some tumor types such as melanoma or hepatocellular carcinoma^23^,^29^, while having no significant effect in colorectal tumors.

Our results indicate that restauration of EPHA3 expression in colon cancer cells with low/undetectable EPHA3 levels, or *EphA3* deletion in mouse models of intestinal tumorigenesis, does not alter the phenotype of these tumor cells. However, several EPH receptors have been shown to have tumor promoting or inhibitory activity in the same tumor type depending on the levels of activation of EPHA3 signaling^42^,^43^,^44^. EPHA3 has recently been shown to be highly expressed in glioblastoma, where it remains kinase-dormant due to reduced or absent Ephrin ligand expression, and plays an important role maintaining glioblastoma cells in an undifferentiated state and sustains rapid proliferation^28^. However, either EPHA3 knockdown or EPHA3 kinase activation through binding to Ephrin A5 or an agonist antibody, resulted in a significant reduction of the growth of glioblastoma cells^28^, suggesting that in these tumors EPHA3 has ligand/kinase-independent oncogenic activity and ligand/kinase-dependent tumor suppressor activity. In colorectal tumors, the mutations observed in EPHA3 have been shown to disrupt its ligand binding capacity or its kinase activity^16^. However, these mutations could result in a gain of function of EPHA3 or preserve kinase-independent oncogenic activity. To investigate this possibility, we overexpressed mutant EPHA3^D806N^ in DLD1 and LS174T colon cancer cells with low endogenous EPHA3 expression, or used a CRISPR/Cas9 strategy to knockout the mutant EPHA3 expressed at high levels in SW48 cells. However, we observed no changes in the motility or proliferation in these isogenic cell line systems, suggesting that in colon cancer cells EPHA3 mutations are not oncogenic. These results seem at odds with recent data suggesting that wild type, as well as mutant EPHA3 may have oncogenic activity in colon epithelial cells^41^. However, a single mouse cell line model was used in this study by Li and collaborators^41^, and further investigation will be required to clarify the role of EPHA3 in the earlier steps of the oncogenic process.

EPHA3 tumor levels have been reported to be associated with the survival of patients with different cancer types, including hepatocellular carcinoma^29^, gastric^30^ and lung^45^ cancer. Interestingly, high EPHA3 protein levels assessed by immunohistochemistry in colorectal tumors have been reported to be associated with poor prognosis of colorectal cancer patients as well as other clinicopathological features of these tumors^31^,^41^. Here, we assessed the levels of EPHA3 in the tumors of a cohort of 159 patients with locally advanced (Dukes C) colorectal cancer by immunohistochemistry with a rabbit polyclonal antibody that specifically detects human EPHA3 on formalin-fixed, paraffin embedded samples (Supplementary Figure 3). Our analysis did not find any associations between EPHA3 tumor levels and disease-free survival, overall survival or any clinicopathological features of Dukes C colorectal cancer patients. This apparent discrepancy could be due to the use of different antibodies to detect EPAH3 in different studies. Alternatively, EPHA3 levels could be associated with tumor stage, as suggested by Xi and Zhao^31^, but have no prognostic value when the analysis is restricted to patients with the same disease stage.

In conclusion, we have investigated here the functional role of EPHA3 on the oncogenic process of colorectal cancer. Although *EPHA3* was reported to be one of the most frequently mutated genes in colorectal tumors, our studies using inducible isogenic cell line systems, mouse models and large human tumor collections, did not reveal a major role of this EPH receptor on proliferation/motility/invasion of cancer cells, tumor initiation/progression/metastasis in mouse models or survival of colorectal cancer patients. This could be due to high levels of redundancy between EPH receptors in colorrectal tumors. Alternatively, *EPHA3* mutations may affect the tumor phenotype in aspects not directly investigated in this study, such as angiogenesis, the immune response of the host or growth under starvation/hypoxic conditions. These results represent an important contribution to our understanding of the role of EPH signaling during colorectal carcinogenesis, and underscore the need for functional studies *in vitro* and *in vivo* to confirm the relevance of putative cancer driver genes identified in sequencing efforts of the cancer genome.

## Material and Methods

### Cell Lines

DLD1 and LS174T colon cancer cell lines were cultured on RPMI medium with 10% fetal bovine serum (Sigma) and 1x antibiotic antimycotic (Life Technologies) at 37 °C and 5% CO_2_. LS174T (*EPHA3* wild type) and DLD1 (heterozygous EPHA3 T719I and M847K mutations) cells carrying the tet-repressor plasmid (TR1 and TR7, respectively) were generated using the T-rex system (Invitrogen) as described previously^46^. To generate EPHA3-inducible clones, DLD1-TR7 and LS174T-TR1 expressing the Tet repressor were transfected with pLenti-CMV/TO-EPHA3 and the corresponding empty vector (pLenti-CMV/TO Neo DEST, Addgene #17292) with Lipofectamine 2000. Transfectants were selected in medium containing G418 (1 or 0.5 mg/ml for DLD1 and LS174T respectively; Invitrogen). Resistant clones were picked and expanded. After doxycycline treatment (1 μg/ml; Sigma) for 48 h, the overexpression of EPHA3 was tested by FACS and western blot. EPHA3^D806N^ was conditionally overexpressed in parental DLD1 and LS174T cells after lentiviral transduction with pINDUCER20-EPHA3^D806N^ or control pINDUCER20-GFP^47^. Viral supernatants obtained from HEK293 cells were applied to the cells in the presence of 8 μg/ml polybrene and stable cultures were selected in medium supplemented with 2 mg/mL G418. After doxycycline treatment (1 μg/mL; Sigma) for 48 h/72 h, the overexpression of EPHA3 (WT or D806N mutant) was tested by FACS and western blot. Cells transfected with pLenti-CMV/TO (empty vector) or pINDUCER20-GFP were used to control for possible non-specific effects of doxycycline treatment.

SW48 colon cancer cells show high levels of EPHA3 expression (Supplementary Figure 2A) and have two mutant alleles of *EPHA3* (K365Nfs*6 and G784E)^18^,^27^ that have also been observed in primary colorectal tumors^48^. A CRISPR/Cas9 approach was used to knockout both EPHA3 alleles in SW48 cells. The sgRNA CTCTGTTCTCGACAGCTTCG was cloned into pSpCas9(BB)-2A-GFP (Addgene #48138). SW48 cells were transiently transfected with pSpCas9(BB)-2A-GFP-sgEPHA3 using Lipofectamine 2000. GFP-positive cells were sorted 48 h post-transfection and plated at low density. After two weeks, individual clones were expanded, DNA extracted, the target region was PCR amplified (EPHA3-Ex1-F: CGG CCT CAT CAC ATC TTC TG and EPHA3-Ex1-R: CTT CGG TGA GAA CGT GCT TT) and sequenced. Three clones with confirmed EPHA3 mutations and loss of EPHA3 expression as well as three EPHA3 wild type clones retaining EPHA3 expression were selected.

### Clinical samples

Samples from colorectal cancer patients with locally advanced disease (Dukes C) were collected at nine hospitals from Finland and informed consent for genetic analysis of the tumor sample was provided by each patient as previously described^49^,^50^. All methods were carried out in accordance with relevant guidelines and regulations. All experimental protocols were approved by the appropriate ethics review committees as described^49^,^50^. The mean follow up of these patients was 7.3 years (range from 3.1 to 9.5 years). For tissue microarray preparation, areas containing a high proportion of tumor cells were selected after histological examination of hematoxylin and eosin stained tumor sections. As previously described^9^,^51^, triplicate 0.6 mm cores from every sample were arrayed in a fresh paraffin block using a Beecher Instrument tissue arrayer (Silver Spring, MD). Unstained 4 μm sections from the tissue microarray were mounted on slides coated with 3-aminopropyl-triethoxy-silane (Sigma, St Louis, MO).

### RNA extraction and quantitative RT-PCR

Total RNA was extracted from mouse liver and intestinal epithelial cells using TRI Reagent according to the manufacturer’s instructions (Molecular Research Center). The RNA (500 ng) was reverse transcribed using the High Capacity cDNA Reverse Transcription kit (Applied Biosystems), and relative *Epha3* mRNA levels were assessed by Real-Time PCR (SYBR Green Master Mix; Applied Biosystems, Branchburg, NJ). 18s rRNA (Taqman Master Mix) was used as a standardization control for the 2^−ΔΔCt^ method as described before^52^. The primers used were *Epha3*-qPCR-F: 5′-CAGCCTTCCAACGAAGTTAAT 3′; *Epha3*-qPCR-R: 5′-CCATGGGATGGGTAGGAG-3′; *18 s* rRNA-F: 5′-AGTCCCTGCCCTTTGTACACA -3′; *18 s* rRNA-R: 5′-GATCCGAGGGCCTCACTAAAC-3′; *18s* Probe: 5′-FAM-CGCCCGTCGCTACCGATTGG-TAMRA-3′.

### Western blotting

Cell cultures were harvested at 70% confluence and cell pellets resuspended in radioimmunoprecipitation (RIPA) assay buffer (0.1% SDS, 1% NP40 and 0.5% Na-deoxycholate in PBS) complemented with protease inhibitors (Pepstatine 5 μg/μl, PMSF 0.3 mM, Aprotinine 1 μg/μl and Sodium orthovanadate 0.1 μM). Aliquots of total protein (100 μg) were loaded on 10% acrylamide gels. After gel electrophoresis, proteins were transferred to a PVDF membrane and probed as described with rabbit anti-Epha3 (1:200; clone L18, Santa Cruz), rabbit anti-EphrinA5 (1:500; Novus Biological), mouse anti-phospho-tyrosine (1:2000; clone PY20, BD Transduction Laboratories) or mouse anti-β-Tubulin (1:2500; clone TUB 2.1; Sigma).

### Xenograft and lung metastasis mouse models

Twelve NOD/SCID mice (Harlan Laboratory) 7/8-week-old were injected subcutaneously with 2.8 × 10^6^ DLD1-EPHA3 cells (right flank) and the control DLD1-EV cells (left flank) resuspended in 100 μl PBS. The animals were randomized in a group receiving doxycycline in the drinking water (1 mg/ml doxycycline and 2.5% sucrose, Sigma) or a control group (2.5% sucrose). The same experimental set-up was carried out for LS174T-EPHA3 and the corresponding empty vector control cells (2.8 × 10^6^cells). Tumor size was measured using a caliper three times per week. Tumor volume was calculated with the formula: V = (L × W2) × 0.5, where L is the length and W is the width of the xenograft. For the model of experimental lung metastasis, LS174T-EPHA3 (3 × 10^6^ cells) or DLD1-EPHA3 (2 × 10^6^ cells) resuspended in 100 μl of PBS were injected in the lateral tail vein of NOD/SCID mice (Harlan Laboratory) 8/9-week-old. The animals were randomized into a group receiving doxycycline in the drinking water (1 mg/ml doxycycline and 2.5% sucrose, Sigma) or a control group (2.5% sucrose) and sacrificed at the indicated time. The number of lung metastatic foci was scored and then the lungs were formalin-fixed, paraffin-embedded, sectioned and stained with hematoxylin and eosin. All animal experiments were carried out under protocols approved by the Vall d’Hebron Ethical Committee for Animal Experimentation and the appropriate governmental agency and carried out in accordance with the approved guidelines.

### Immunoprecipitation

Cells were lysed with RIPA buffer containing protease and phosphatase inhibitors and briefly sonicated. Total protein (1 mg) was pre-incubated with mouse anti-EPHA3 (clone IIIA4^28^; 1 μg per 1 mg of protein) in immunoprecipitation (IP) buffer (50 mM Tris-HCl pH7.5, 150 mM NaCl, 1.5 mM MgCl_2_, 1% Triton X-100, 5% glycerol) at 4 °C overnight. Prewashed protein G-Agarose beads (Santa Cruz Biotechnology) were added with further incubation for 1 h at 4 °C. Samples were then washed five times in IP buffer and analyzed by western blotting with the indicated antibodies.

### FACS analysis

Cell surface expression of EPHA3 (WT or D806N mutant) in LS174T and DLD1 engineered cell lines was confirmed by FACS. Cells (5 × 10^5^ per sample) pretreated with doxycycline for 48 h/72 h were suspended in 100 μl of PBS with 5 μg/ml anti-EPHA3 (clone IIIA4) or anti-GAPDH (clone 6C5; Santa Cruz Biotechnology) for 1 h. Cells were washed with cold PBS and incubated with FITC or CFL647-labeled secondary antibodies specific for mouse IgG (114-096-062; Jackson Immuno Research Laboratories and sc-362287; Santa Cruz Biotechnology, respectively). After incubation for 30 min at 4 °C, cells were again washed and resuspended in PBS-propidium iodide solution (2 μg/ml). Fluorescence was quantified with a BD FACSCalibur instrument and CellQuest Software (BD Biosciences).

### Mouse knockout strains and azoxymethane treatment

*Apc*^*min/+*^ mice on a 129/Sv background have been described before^53^. These mice carry a heterozygous mutation in *Apc* inducing formation of multiple polyps mainly in the small intestine^21^. The *EphA3* knockout mouse has been previously described and is on a 129 × 1/SvJ genetic background^19^. The first exon of *EPHA3,* encoding its signal sequence, was removed and replaced with a PGK-neo cassette by homologous recombination. Male *Apc*^*min/+*^;*EphA3*^*+/+*^ mice were crossed with female *Apc*^*+/+*^;*EphA3*^*−/−*^ mice to obtain *Apc*^*min/+*^;*EphA3*^*+/−*^ males and *Apc*^*+/+*^;*EphA3*^*+/−*^ females that were subsequently crossed to obtain the experimental animals *Apc*^*min/+*^;*EphA3*^*+/+*^, *Apc*^*min/+*^;*EphA3*^*+/−*^ and *Apc*^*min/+*^;*EphA3*^*−/−*^. In addition, nine-week-old *EphA3*^*+/+*^ and *EphA3*^*−/−*^ (both *Apc*^*+/+*^) mice were i.p. injected with the intestine-specific carcinogen azoxymethane (AOM; 10 mg/kg; Sigma) weekly for 9 weeks and sacrificed 7 weeks after the last AOM injection.

### Histology and immunohistochemistry

Eighty-six-week-old mice (*EphA3*^*+/+*^ and *EphA3*^*−/−*^) or forty-one-week-old mice (*Apc*^*min/+*^;*EphA3*^*+/+*^, *Apc*^*min/+*^;*EphA3*^*+/−*^ *and Apc*^*min/+*^;*EphA3*^*−/−*^) were sacrificed, the small and large intestines were dissected, opened longitudinally and fixed with 4% formalin. Tumor size and number were scored under a dissecting microscope (OLYMPUS SZH stereo-zoom microscope, magnification X7.5) before paraffin inclusion. The intestine was rolled longitudinally using the ‘Swiss roll’ technique^54^ and embedded in paraffin. For immunohistochemistry, the NovoLink polymer detection system (Novocastra Laboratories) was used. Human EPHA3 immunostaining was carried out with a rabbit polyclonal anti-EPHA3 (1:200; Clone L18, Santa Cruz). EPHA3 staining levels were scored using a semiquantitative scale from 0 (absence of EPHA3 Immunostaining) to 3 (highest immunostaining). EPHA3 expression was evaluated blinded from the clinical data. For Kaplan-Meyer plots, EPHA3 levels were dichotomized as low or high EPHA3 using an average score cutoff value of 1.5. Importantly, no significant survival differences between high/low EPHA3 groups were observed with any other possible cutoff value. EPHA3 was considered as a continuous variable for Cox multivariate regression analysis of prognostic factors for these patients (covariates: EPHA3 levels, histologic grade, sex, age, and tumor location) as show in Supplementary Table 1.

### Clonogenicity assay

LS174T-EPHA3 or DLD1-EPHA3 cells and the corresponding empty vector control cells were seeded (5 × 10^2^) into 6-well plates and allowed to attach overnight. The medium was then replaced with complete medium with or without doxycycline (1 μg/ml) as indicated and cells allowed to grow for 10 days. Plates were then stained with crystal violet 0.1% and the number of macroscopically visible colonies was scored blinded from the sample identity. Three independent experiments were carried out in triplicate.

### *In vitro* proliferation assay

Cells were seeded into 24-well plates in triplicate and allowed to attach overnight (5 × 10^5^ for LS174T-EPHA3 or 3 × 10^5^ for DLD1-EPHA3 and the corresponding empty vector cells). Doxycycline (1 μg/ml) was added as indicated. Cell counting was performed by cell trypsinization and staining with trypan blue. Viable cells were counted using a hemocytometer at times 0, 24, 48, 72 and 96 h. Growth curves presented are the average of three independent experiments carried out in triplicate.

### Wound-healing assay

Cells were seeded into 6-well plates (2 × 10^6^ cells per well) and allowed to grow until they reached 90% confluence with or without doxycycline (1 μg/ml) as indicated. The cell monolayer was scratched with a sterile micropipette tip and the wound region was allowed to heal by cell migration. The area that remained clear of cells after 4, 8, 12, 24 and 48 h was quantified blinded from sample identity with Image J (National Institutes of Health, NIH) and compared with the area of the wound at time zero. The average of three independent experiments in triplicate is shown.

### Matrigel invasion assay

The ability of cells to invade through matrigel-coated filters was determined using a 24-well Boyden chamber (Beckton Dickinson; 8 μm pore size) covered with 100 μl of 1 mg/mL Matrigel (Beckton Dickinson). Cells (6 × 10^5^ of LS174T-EPHA3 or 3 × 10^5^ of DLD1-EPHA3) were seeded in 100 μl of RPMI medium containing 1% FBS in the upper compartment of the transwell. Where indicated doxycycline (1 μg/ml) was added. The lower compartment was filled with RPMI medium (with or without doxycycline) containing 10% FBS, acting as an attractant. After incubation for 48 h at 37 °C in 5% CO_2_, the cells that did not penetrate the filter were wiped out with a cotton swab, and the cells that had invaded into the lower surface of the filter were fixed and stained with 5% crystal violet. Filters were mounted on microscope slides to enable cell counting under the microscope (10X) blinded from the sample identity. The total number of invading cells was determined and the average of three independent experiments run in triplicate is shown.

### Soft-agar colony formation assay

LS174T-EPHA3 or DLD1-EPHA3 cells were resuspended (1 × 10^5^) in complete RPMI medium containing 0.3% agar with or without doxycycline (1 μg/ml) and then plated into 6-well plates on top of 0.6% agar in RPMI medium. Cultures were maintained at 37 °C in a 5% CO_2_ incubator for 2–3 weeks depending on the cell line. Fresh complete RPMI medium was added with or without doxycycline (1 μg/ml) every 2–3 days. The colonies were stained with nitro blue tetrazolium chloride (1 mg/ml; Sigma) and the number of macroscopically visible colonies was scored blinded from the sample identity. Three independent experiments were carried out in triplicate.

## Additional Information

**How to cite this article**: Andretta, E. *et al*. Investigation of the role of tyrosine kinase receptor EPHA3 in colorectal cancer. *Sci. Rep.* **7**, 41576; doi: 10.1038/srep41576 (2017).

**Publisher's note:** Springer Nature remains neutral with regard to jurisdictional claims in published maps and institutional affiliations.

## Supplementary Material

Supplementary Materials
