# Supplementary material for: Investigation of the role of tyrosine kinase receptor EPHA3 in colorectal cancer
Source: Sci Rep. 2017 Feb 7;7:41576. doi: 10.1038/srep41576 (PMC5294649; doi:10.1038/srep41576)

Investigation of the role of tyrosine kinase receptor EPHA3 in colorectal cancer

Elena Andretta[1], Fernando Cartón-García[1], Águeda Martínez-Barriocanal[1], Priscila Guimarães de Marcondes[1], Lizbeth M. jimenez-Flores[1], Irati Macaya[1], Sarah Bazzocco[1], Josipa Bilic[1], Paulo Rodrigues[1], Rocio Nieto[1], Stefania Landolfi[2], Santiago Ramon y Cajal[2], Simo Schwartz Jr.[3,4], Arthur Brown[5], Higinio Dopeso[1], Diego Arango[1].

[1]Group of Biomedical Research in Digestive Tract Tumors, CIBBIM-Nanomedicine, Vall d'Hebron University Hospital, Research Institute (VHIR), Universitat Autònoma de Barcelona, Passeig Vall d'Hebron, 119-129, 08035 Barcelona, Spain; [2]Department of Pathology, Vall d'Hebron Hospital, Barcelona, Spain; [3]Group of Drug Delivery and Targeting, CIBBIM-Nanomedicine, Vall d'Hebron University Hospital, Research Institute (VHIR), Universitat Autònoma de Barcelona, Barcelona, Spain; [4]CIBER de Bioingeniería, Biomateriales y Nanomedicina (CIBER-BBN), Spain; [5]Robarts Research Institute, London, Ontario, Canada.

RUNNING TITLE: EPHA3 and colorectal cancer.

Supplementary Figure 1

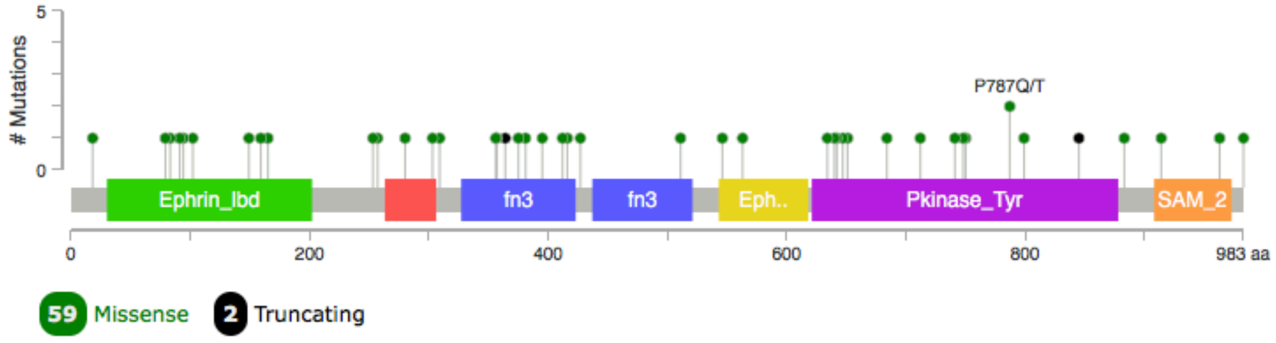

Supplementary Figure 1: EPHA3 mutations reported in colorectal tumors. Representation along the EPHA3 protein of all mutations found in the 1,035 colorectal tumors investigated in these studies: Cell Rep. 2016 Apr 14. pii: S2211-1247(16)30364-3; Nature. 2012 Aug 30;488(7413):660-4; Nature. 2012 Jul 18;487(7407):330-7; and Genome Biol. 2014 Aug 28;15(8):454. Figure generated with MutationMapper (cbioportal.org).

Supplementary Figure 2

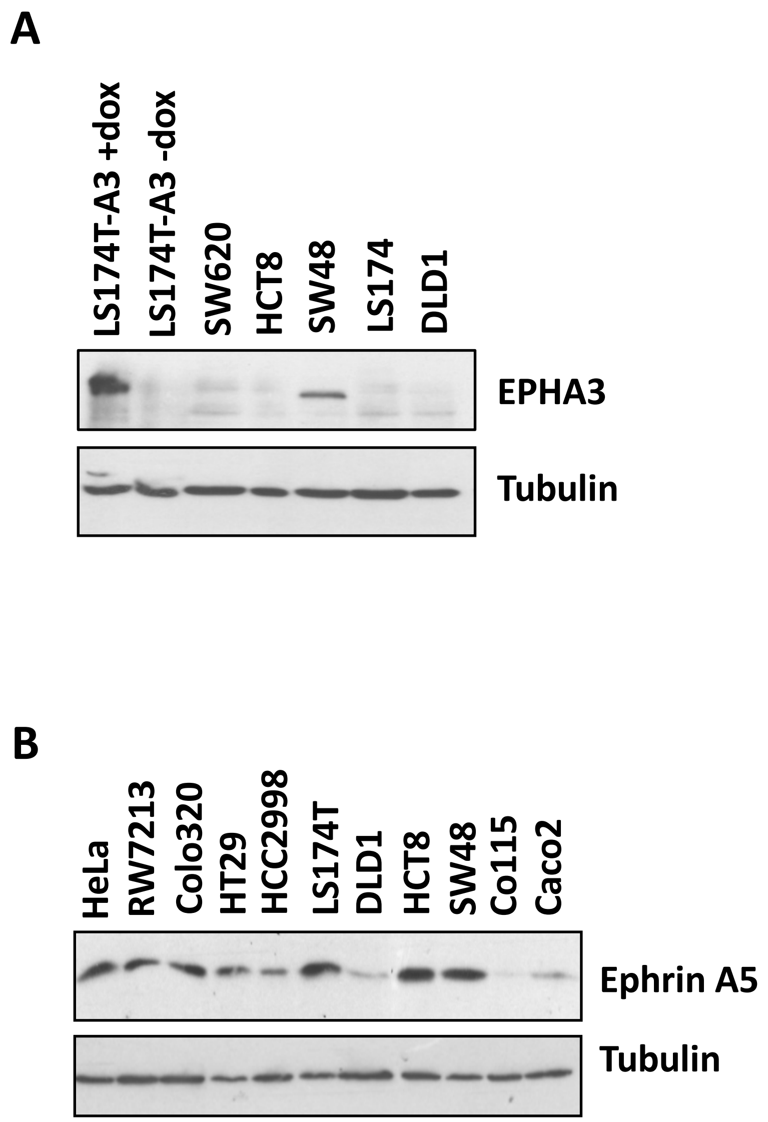

Supplementary Figure 2: EPHA3 and Ephrin A5 levels in colorectal cancer cell lines. The levels of EPHA3 (**A**) and its preferred ligand Ephrin A5 (**B**) were assessed in a panel of colon cancer cell lines by Western blotting. Tubulin levels were used as a loading control.

Supplementary Figure 3

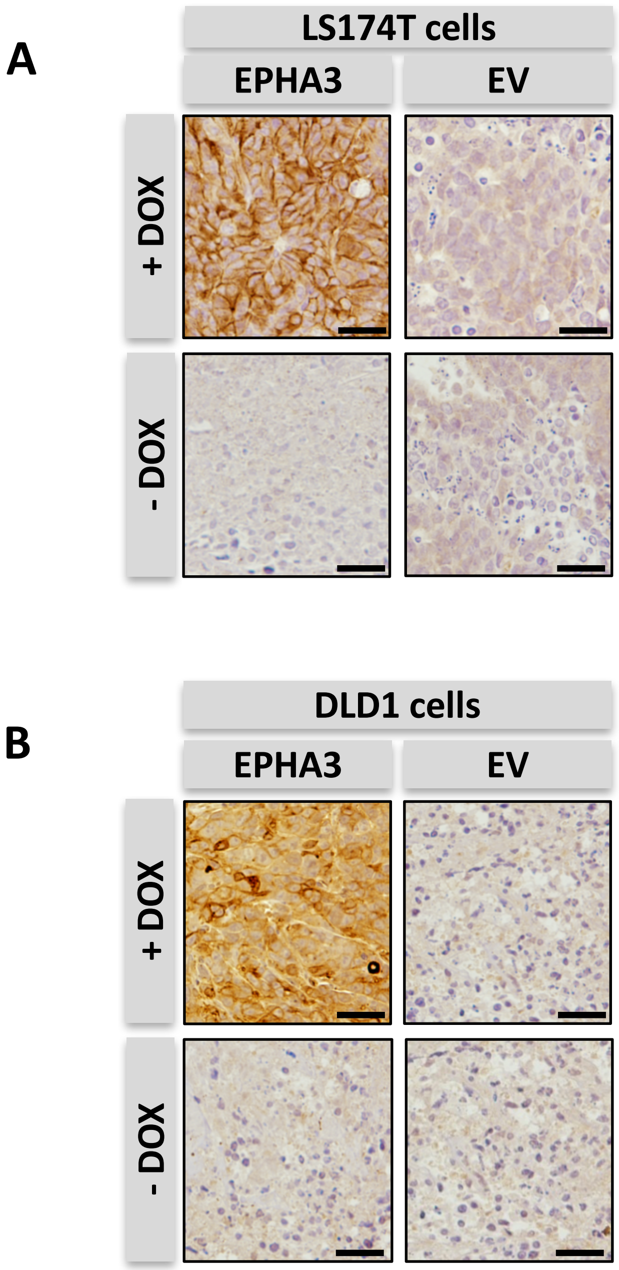

Supplementary Figure 3: EPHA3 levels in xenografts of colon cancer cell lines. The levels of EPHA3 protein expression were determined by immunohistochemistry in formalin-fixed, paraffin-embedded samples from the subcutaneous xenografts of LS174T-EPHA3 (**A**) and DLD1-EPHA3 (**B**) cells grown in NOD/SCID mice that received doxycycline (Dox) in the drinking water, or in control mice with no doxycycline. EPHA3 levels were also assessed in the corresponding derivative cell lines transfected with the control empty vector. Scale bar: 50µm.

Supplementary Figure 4

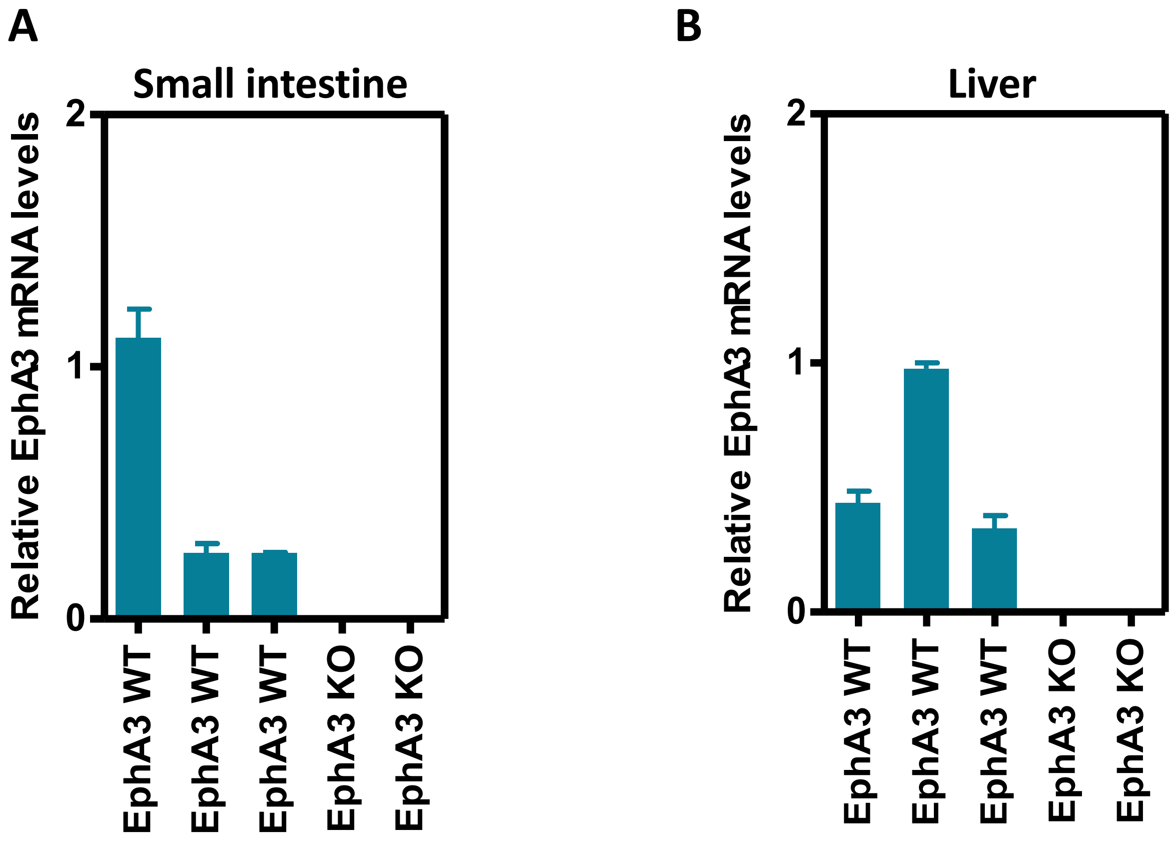

Supplementary Figure 4: EPHA3 levels in wild type and knockout mice. The levels of EphA3 mRNA were assessed in the small intestine (**A**) and the liver (**B**) of wild type and knockout *EphA3* mice by quantitative Real-Time RT-PCR.

Supplementary Figure 5

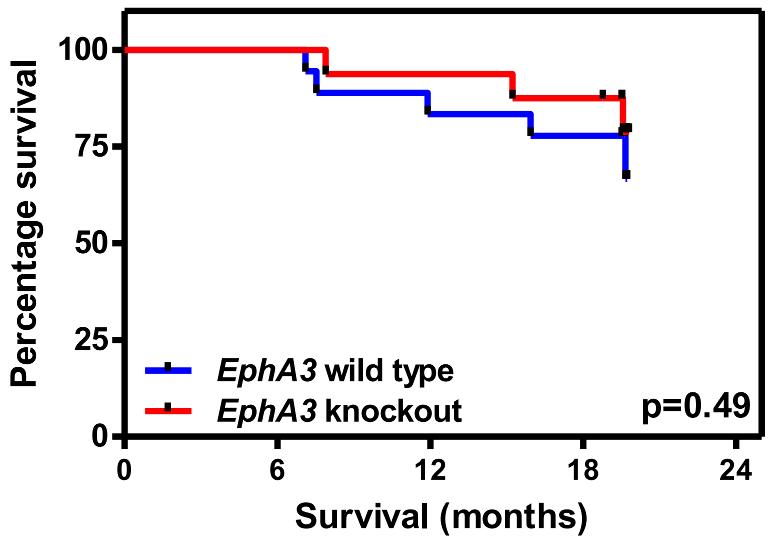

Supplementary Figure 5: Survival of *EphA3* wild type and knockout mice. The percentage of mice alive at different times is shown for *EphA3* wild type and knockout animals. The p value was calculated with the Logrank test.

Supplementary Figure 6

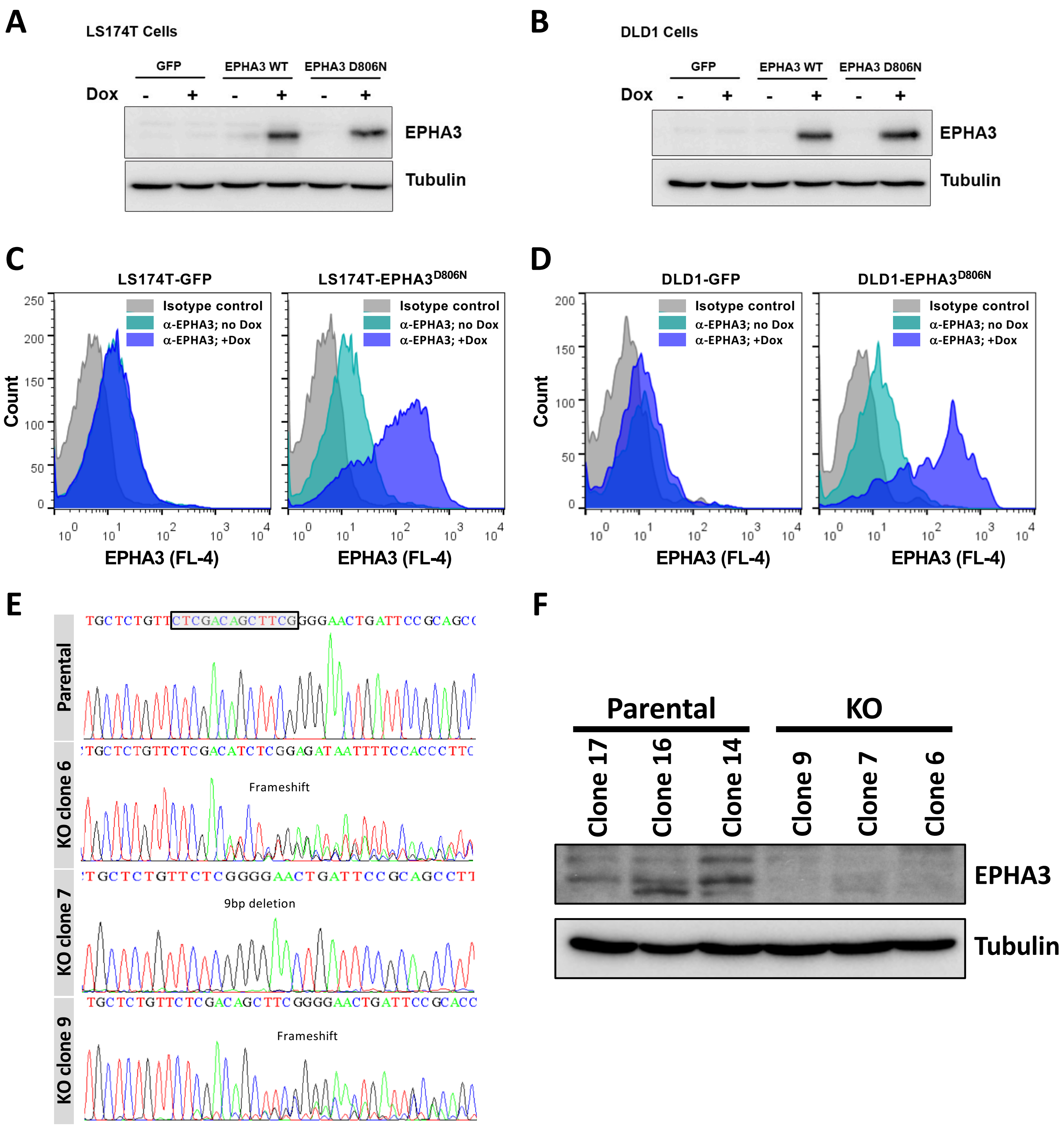

Supplementary Figure 6: Validation of isogenic *in vitro* systems. (**A-B**) Western blot showing the levels of EPHA3 protein in LS174T (**A**) and DLD1 (**B**) cells stably transfected with inducible wild type EPHA3, EPHA3^{D806N} or GFP control after treatment with 1μg/ml doxycycline for 72h. Tubulin levels are shown as a loading control. (**C-D**) Cell surface levels of EPHA3^{D806N} following induction with doxycycline (1μg/ml; 72h) were assessed by flow cytometry analysis in LS174T-EPHA3 (**C**) and DLD1-EPHA3 (**D**). (**E**) Results of Sanger sequencing of the region of endogenous EPHA3 in SW48 cells confirming the presence of truncating mutations in the clones used in this study. Greyed sequence in the parental sequence (CTCGACAGCTTCG) shows the region targeted by the sgRNA used. (**F**) Western blot showing the levels of EPHA3 protein in clones of SW48 single cell clones with (KO) or without (parental) CRISPR/Cas9 targeted EPHA3 mutations.

Supplementary Table 1: Clinicopathological features of the 159 Dukes C colorectal cancer patients in this study.

	All cases	Low EPHA3	High EPHA3	p Value
**Sex, n (%)**				
**Female**	99	53 (54.1)	25 (43.1)	0.24[1]
**Male**	58	45 (45.9)	33 (56.9)	
**Age (years), mean±SD**	64.69±12.93	65.2±13.6	63.8±11.9	0.36[2]
**Site, n (%)**				
**Colon**	39	57 (58.1)	38 (64.4)	0.78[1]
**Rectum**	29	41 (41.9)	21 (35.6)	
**Degree of differentiation, n (%)**				
**Good**	19	12 (12.4)	7 (12.3)	0.99[1]
**Moderate**	114	72 (74.2)	42 (73.7)	
**Poor**	21	13 (13.4)	8 (14.0)	
**Mean Follow up, mean±SD**	7.3±1.1	7.2±1	7.5±1.2	0.17[3]
**Adjuvant treatment, n (%)**				
**Yes**	70	41 (41.8)	29 (49.2)	0.4[1]
**No**	87	57 (58.2)	30 (50.8)	
**5- year overall survival, n (%)**				
**Alive**	61	38 (38.4)	23 (38.3)	1[1]
**Dead**	98	61 (61.6)	37 (61.7)	
**5- year disease-free survival, n (%)**				
**Alive**	67	40 (41.7)	27 (46.6)	0.61[1]
**Dead**	87	56 (58.3)	31 (53.4)	

[1]Fisher test; [2]Mann Whitney test; [3]Student's T-test.

Figure 1 – FULL LENGTH BLOTS

Original Figure

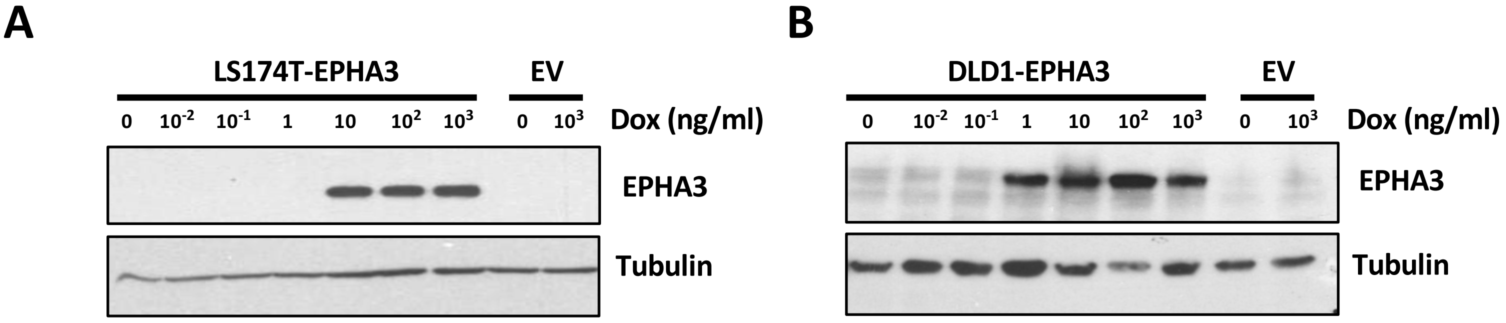

Full length blots

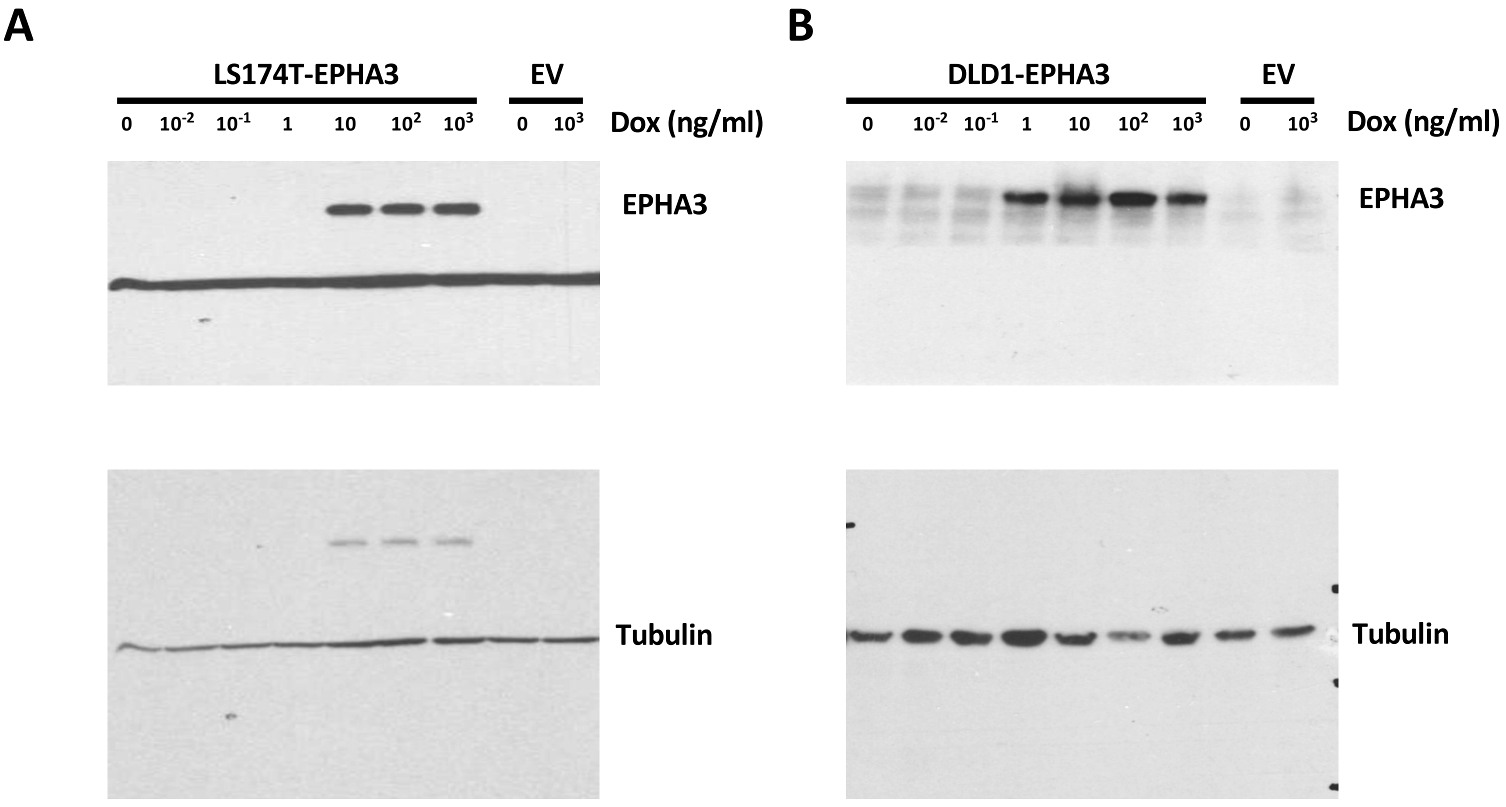

Supplementary Figure 2 – FULL LENGTH BLOTS

Original Figure

A

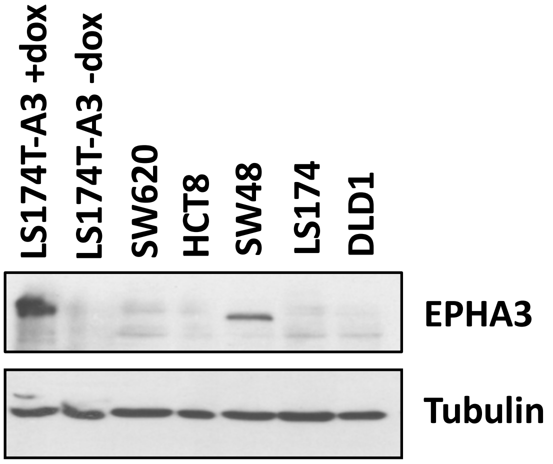

B

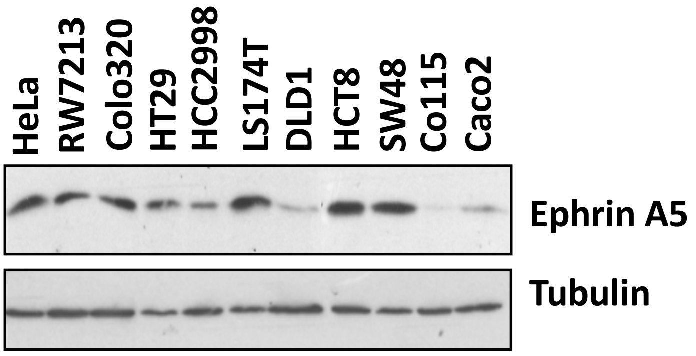

Full length blots

A

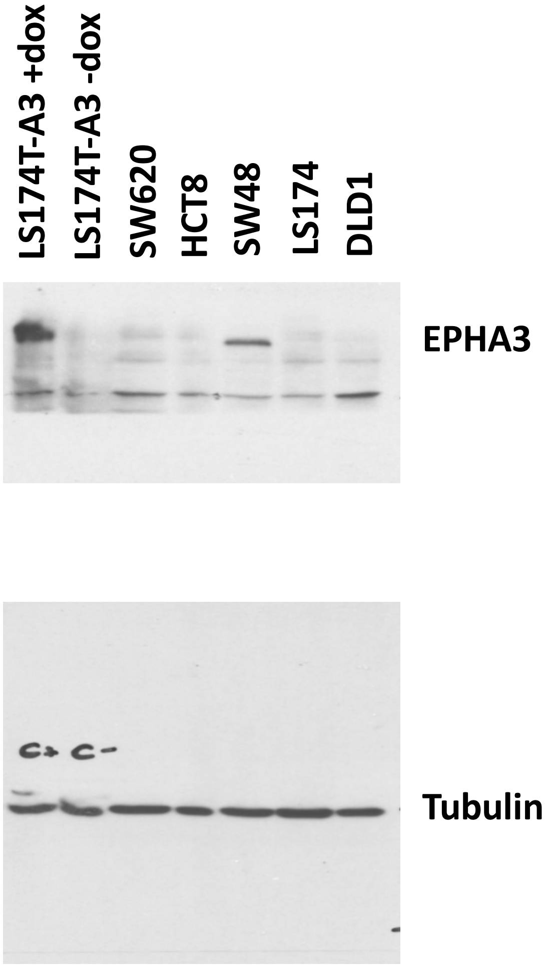

B

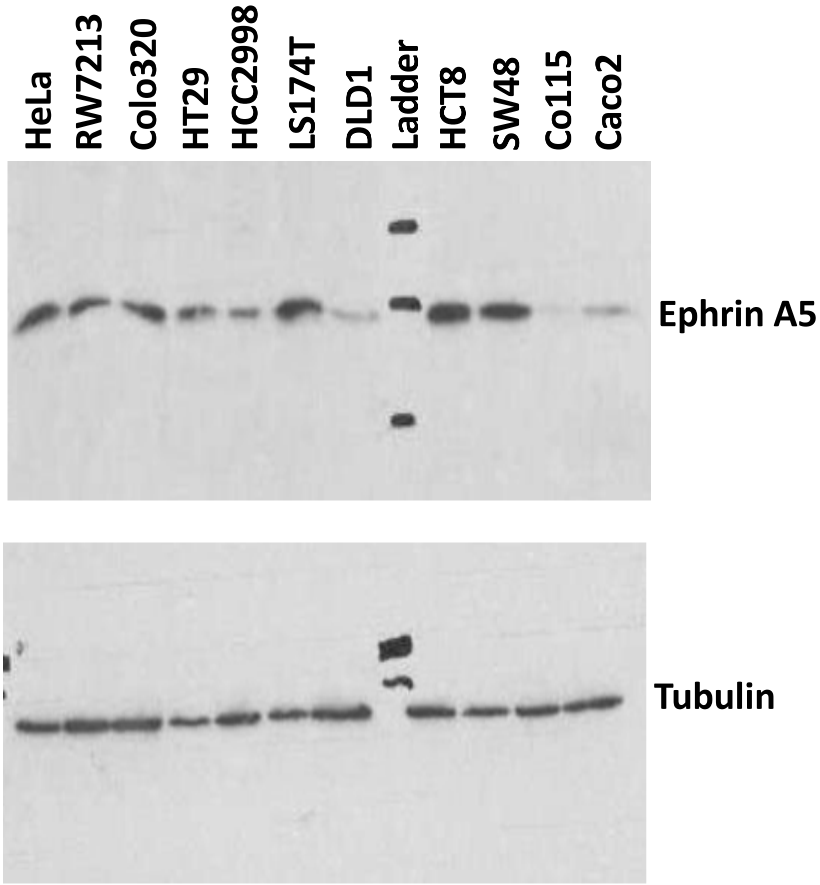

Supplementary Figure 6 – FULL LENGTH BLOTS

Original Figure

A

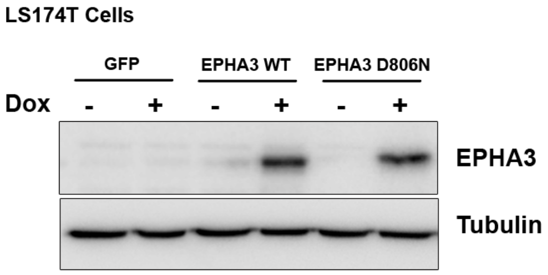

B

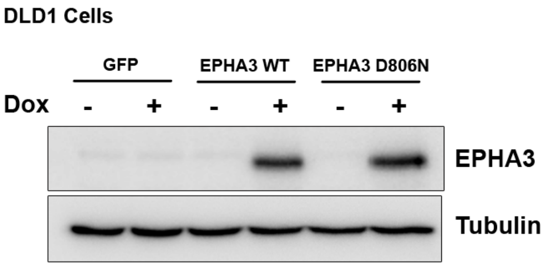

Full length blots

A

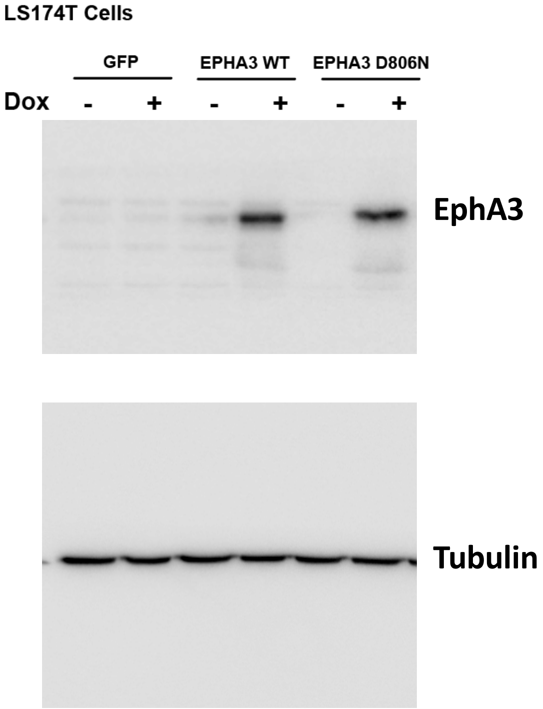

B

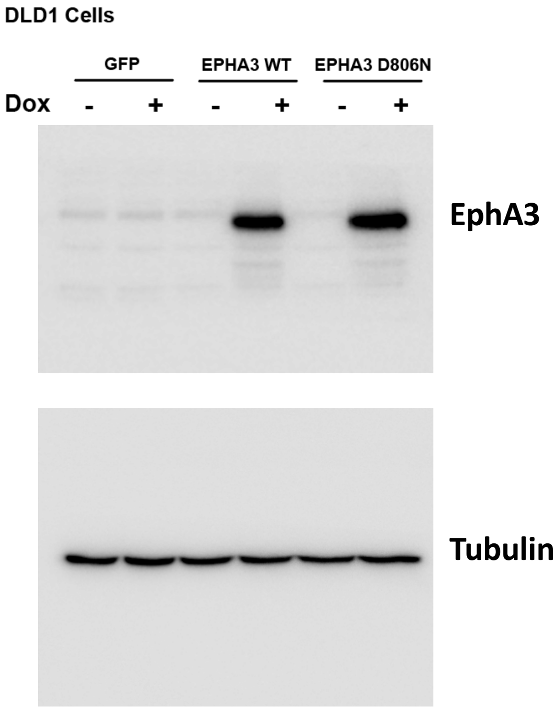

Supplementary Figure 6 – FULL LENGTH BLOTS

Original Figure

F

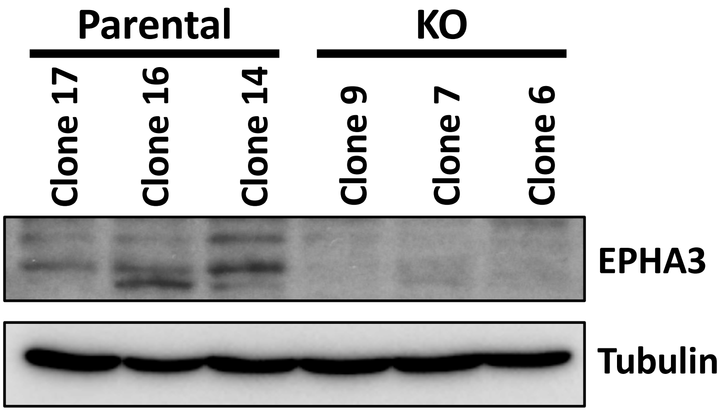

Full length blots

F

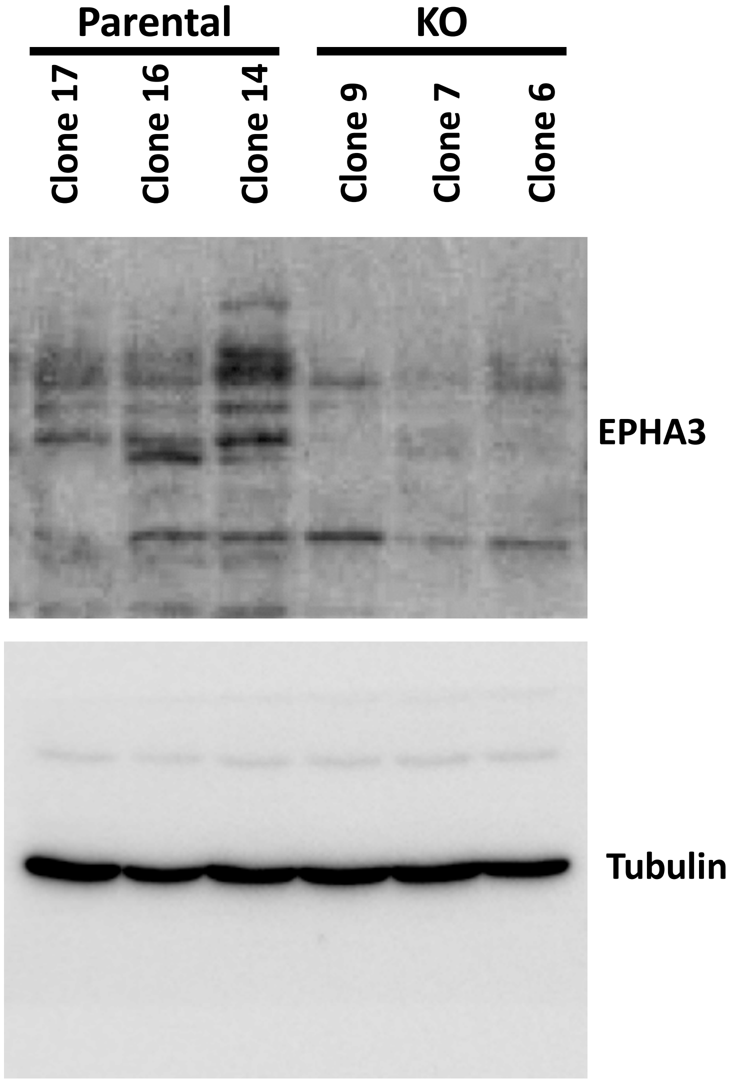

Supplement: Supplementary Materials [file srep41576-s1.pdf]
